# Can Gaming Get You Fit?

**DOI:** 10.3389/fphys.2020.01017

**Published:** 2020-08-20

**Authors:** Jonathan Berg, Alf Inge Wang, Stian Lydersen, Trine Moholdt

**Affiliations:** ^1^Department of Circulation and Medical Imaging, Norwegian University of Science and Technology, Trondheim, Norway; ^2^St. Olav’s University Hospital, Trondheim, Norway; ^3^Department of Computer Science, Norwegian University of Science and Technology, Trondheim, Norway; ^4^Regional Centre for Child and Youth Mental Health and Child Welfare, Norwegian University of Science and Technology, Trondheim, Norway

**Keywords:** active video games, cardiorespiratory fitness, exercise training, gamification, high-intensity interval training, physical activity

## Abstract

**Rationale:**

Exergaming may be a viable alternative to more traditional exercise. As high-intensity exercise can provide substantial health benefits, the purpose of this study was to investigate the long-term effectiveness of providing inactive adults with access to a high-intensity exergaming platform.

**Methods:**

In this study, 52 inactive adults (<150 min of exercise per week), aged 18 years or older, were randomized (1:1) into an exergaming (*N* = 27) or a control group (*N* = 25). Exergaming participants were given access to the Playpulse exergaming platform for 6 months, where they decided how frequently they wanted to use the platform. The primary outcome measure, analyzed with a mixed model, was peak oxygen uptake (V̇O_2peak_). Secondary outcomes included body composition, blood pressure, and blood markers of cardiometabolic health.

**Results:**

Mean V̇O_2peak_ at 6 months was 42.3 (SD 7.0) mL⋅kg^−1^⋅min^−1^ and 41.9 (SD 7.4) mL⋅kg^−1^⋅min^−1^ for the exergaming and control group, respectively with no significant between-group differences (-0.7, 95% CI -2.7 to 1.3, *P* = 0.49). Apart from increased moderate-intensity physical activity in the exergaming group at 3 months (21.9 min⋅day^−1^, 95% CI: 2.2 to 41.5, *P* = 0.03) compared to the control group, there were no significant between-group differences for any outcome at either 3 or 6 months. On average, participants in the exergaming group performed 12 (SD 13) exergaming sessions with an average heart rate of 74.5 (SD 7.5)% of maximum heart rate, throughout the intervention.

**Conclusion:**

Due to low exergaming frequency over the 6-month intervention, exergaming participants showed no significant health benefits compared to control. Our study indicates that although the Playpulse exergaming platform is found enjoyable, this is not enough to motivate inactive adults to regularly engage in exercise and thereby improve health.

**Clinical Trial Registration:**

www.clinicaltrials.gov, identifier NCT03513380.

## Introduction

Physical inactivity and sedentary behavior are independently associated with all-cause mortality (Lee et al., 2012). With the average Norwegian adult spending 60% of his/her awake time in sedentary behavior, and only one out of three adults fulfilling the current recommendations for physical activity (Hansen et al., 2015), there is a great need to increase physical activity levels, decrease sedentary time, and/or counteract the detrimental effects associated with these behaviors. High cardiorespiratory fitness is associated with a lower risk of all cause-mortality and is suggested to be more important for survival than physical activity *per se* (Kodama et al., 2009; Lee et al., 2011; Davidson et al., 2018). Also, an increase in cardiorespiratory fitness eliminates the deleterious effects of sedentary behavior, even among those who are insufficiently active (Nauman et al., 2016). Consequently, interventions aiming to improve health outcomes should emphasize improvements in cardiorespiratory fitness. Compared to moderate-intensity exercise, exercise with higher intensity requires less time to achieve the same or even superior health benefits (Helgerud et al., 2007; Nes et al., 2012). However, compliance is necessary and successful long-term interventions outside controlled environments are lacking, likely due to low adherence (Williams et al., 2007; Gray et al., 2016; Roy et al., 2018). Thus, alternatives to traditional exercise that promote independent exercise behavior are warranted. An important variable for exercise participation and adherence is enjoyment (Salmon et al., 2003; Williams et al., 2008). In an endeavor to increase the attractiveness of exercise, the concept of active video gaming, also called exergaming has emerged (Oh and Yang, 2010). Exergaming is an enjoying alternative to traditional exercise and may generate greater adherence (Graves et al., 2010; Monedero et al., 2015; Moholdt et al., 2017). Prior studies in adults have primarily focused on the acute effects of exergaming where most have reported light-to-moderate exercise intensities, although higher exercise intensities have been reported (Graves et al., 2010; Peng et al., 2011; Moholdt et al., 2017; McBain et al., 2018; Viana et al., 2018; Naugle et al., 2019; Berg and Moholdt, 2020). Even if a recent systematic review suggested that exergaming may confer health benefits, the authors highlighted that the long-term effectiveness of exergaming is unknown (Street et al., 2017). Furthermore, previous studies have been limited by assessing exergames that at most can elicit moderate exercise intensity (Street et al., 2017; Bock et al., 2019).

Therefore, this study aimed to investigate if 6 months’ access to a vigorous intensity exergaming platform could provide health benefits for inactive adults. In addition, we aimed to assess exergaming frequency, exercise intensity, and enjoyment over time in the exergaming group. We hypothesized that after 6 months, (1) the exergaming group would improve peak oxygen uptake (V̇O_2peak_) more than the control group, (2) the exergaming group would be more physically active than the control group, and (3) the exergaming group would show greater improvement in body composition and markers of cardiometabolic health compared to the control group.

## Materials and Methods

### Design

This study was undertaken at the Norwegian University of Science and Technology (NTNU) and St. Olav’s University Hospital in Trondheim, Norway. The Regional Committee for Medical and Health Research Ethics in Central Norway approved the study (2017/1501), and written informed consent was obtained from all participants before participation. The study was registered in the ClinicalTrials.gov registry (NCT03513380). If not otherwise reported, assessments were conducted at baseline (before randomization), halfway through the treatment (3 months), and after the end of treatment (6 months).

### Participants

Inclusion criteria were age ≥18 years, inactive (<150 min of exercise per week), and able to ride a bike for up to 60 min. Participants were excluded if they had any known cardiovascular disease or were taking beta-blockers and/or anti-arrhythmic drugs. We recruited participants via advertisements on the web pages of St. Olav’s University Hospital and NTNU, as well as through social media channels. Participants were enrolled between June and November 2018.

### Interventions

Participants were randomly allocated to an exergaming or a control group. The exergaming group was given access to the exergaming platform “Playpulse” for 6 months (detailed below). There were no mandatory exergaming sessions; instead, the participants chose themselves how often they wanted to use the exergaming platform and scheduled their exergaming sessions via an online calendar. All exergaming sessions were supervised, and participants wore heart rate monitors (Polar A300/H10, Polar, Kempele, Finland) for all sessions. Heart rate data was stored in an online training diary (PolarFlow) for use in analyses of exercise intensity. In addition, following each exergaming session, all participants filled out three separate questionnaires; the Physical Activity Enjoyment Scale (PACES), the Feelings Scale, and a modified 0–10 Borg-Scale, to assess perceived enjoyment and exertion (Hardy and Rejeski, 1989; Kendzierski and DeCarlo, 1991). Furthermore, exergaming frequency and duration were registered. Participants in the control group had no access to the exergaming platform during the 6-month intervention period but were not discouraged from being physically active.

### The Playpulse Exergaming Platform

The Playpulse platform offers several exergames played on a regular stationary bike. In Playpulse, forward propulsion of the player’s feet on the bike causes forward movement in the games, whereas buttons on the handlebars are used to change direction and perform other actions, such as shooting. The structure of the games is designed to motivate the player to perform exercise with high intensity. The most popular and extensive Playpulse exergame is Pedal Tanks, a multiplayer exergame based on teamwork and competition. In the game, a team of two players competes against another team, intending to move a tank, shoot other players’ tanks, take the enemy’s flag, and return their flag to own base. The game has been designed and developed to have similar characteristics as most popular current online games with competitiveness, collaboration, variation in player classes, and a deep, persistent progression system (Hagen et al., 2015, 2016). These characteristics result in varied, enjoyable gameplay that evolves according to the gameflow model (Sweetser and Wyeth, 2005) and produces the right balance between the game’s attractiveness and effectiveness of exercise (Sinclair et al., 2007). The Playpulse games and platform have continuously been evolving from 2015, until today.

### Outcomes

Our primary outcome was cardiorespiratory fitness measured as maximal oxygen uptake (V̇O_2max_). V̇O_2max_ was assessed on a treadmill (Woodway, Waukesha, WI) (Lode, Groningen, Netherlands) using an incremental test to exhaustion. Due to injuries, two participants completed the incremental test on a cycle ergometer. We individually adjusted the start-level to bring participants to volitional exhaustion in 8–12 min. We increased speed/incline or work rate every minute by 1 km⋅h^−1^/2% (treadmill) or 10–30 W (cycle ergometer) each minute until volitional failure. We continuously measured and recorded ventilatory variables with a MetaLyzer 3B system (Cortex, Leipzig, Germany). Criteria for the attainment of V̇O_2max_ was adjusted for age. However, since not all participants fulfilled to criteria for attainment of V̇O_2max,_ we report this as V̇O_2peak_ (Wagner et al., 2020). We report V̇O_2peak_ as the mean of the three highest 10-s values during the test.

Secondary outcomes were physical activity level, body composition, blood pressure, and blood markers of cardiometabolic health. Physical activity was measured using activity monitors (SenseWear, BodyMedia, Pittsburgh, Pennsylvania, United States) worn for 7 days. Objectively measured physical activity is reported as average daily means and categorized in moderate- (3.0–6.0 metabolic equivalents (METs), vigorous- (6.0–9.0 METs), very vigorous- (>9.0 METs) and moderate-to-very vigorous-intensity physical activity (>3.0 METs). Bodyweight and body composition were estimated using bioelectrical impedance analysis (InBody 720/770, Biospace, Seoul, South Korea). Body mass index (BMI) was calculated as weight in kilograms divided by the square of height in meters. After 10 min of supine resting, systolic and diastolic blood pressure and resting heart rate were measured in a seated position using an automatic blood pressure device (Philips IntelliVue MP50, Philips Medizin Systeme, Boeblingen, Germany). We measured blood pressure three times with 2-min intervals, and the mean of the three measurements is reported. At both baseline and after 6 months, participants arrived at the laboratory after ≥10-h fast, and a peripheral intravenous catheter was inserted into a forearm vein. Participants underwent an oral glucose tolerance test in which they drank 75 g of glucose dissolved in 2.5 dl of water. Blood was drawn before and at 30-min intervals for 2 h after the ingestion of glucose. Blood drawn in the fasted state was analyzed for glucose, triglycerides, high-sensitive C-reactive protein (hs-CRP), low-density lipoprotein cholesterol (LDL), high-density lipoprotein cholesterol (HDL) and total cholesterol. Blood samples following the ingestion of glucose were analyzed for glucose. All blood analyses were done immediately at St. Olav’s University Hospital, Trondheim, according to standard procedures.

### Sample Size

We calculated the sample size based on the primary outcome measure (V̇O_2peak_). To provide a power of 0.90, 5% level of significance, and to detect a clinically significant difference of 3.0 mL⋅min^−1^⋅kg^−1^ with a standard deviation of 3.0 mL⋅min^−1^⋅kg^−1^ and using an independent samples-test, we needed 21 participants in each group. To allow for an anticipated drop-out of 15%, we aimed at including a minimum of 50 participants.

### Randomization

After baseline testing, the participants were stratified according to sex and randomly allocated in a 1:1 manner to the exergaming or control group. Randomization was done in blocks of varying sizes using a computer random number generator developed and administrated at the Unit for Applied Clinical Research, NTNU.

### Statistical Methods

We used mixed models with the outcome variables, one at a time, as the dependent variable, participant as random effect, and time point and group and their interaction as categorical covariates. We assumed no systematic effect of group at baseline, as recommended by Twisk et al. (2018). A linear mixed model includes all subjects with data from at least one time point. It is unbiased under the missing at random assumption, while a complete case analysis using data only from subjects with data at all time points is generally unbiased only under the more restrictive missing completely at random assumption (Ashbeck and Bell, 2016). We checked normality of the residuals by visual inspection of Q-Q plots and log-transformed variables that did not show normality before analysis. Since analyses of log-transformed data did not alter the main findings, we report the analyses based on untransformed data. Besides, we also analyzed the association between exergaming frequency and baseline data for the participants allocated to exergaming using multiple linear regression. Furthermore, we used multiple linear regression to assess to what degree exergaming frequency, duration, and/or intensity could explain changes in both primary and secondary outcomes at 6 months. For missing items on the PACES questionnaire, we used the mean scores from the other items when at least 11 items had been answered. Data are reported as mean (standard deviations). All analyses were performed using SPSS 26.0, and the level of significance was set at 0.05.

## Results

**FIGURE 1:**
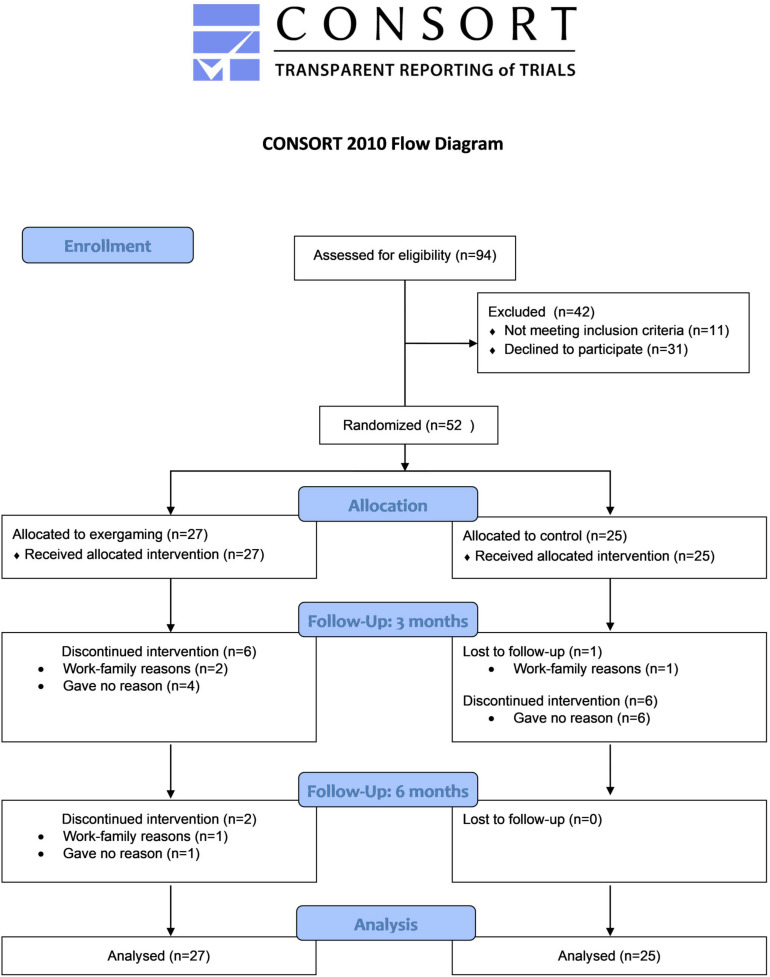
CONSORT flowchart for the present study. Consolidated Standards of Reporting Trials (CONSORT).

Fifty-two inactive adults were recruited and randomly allocated to either exergaming (*n* = 27) or control (*n* = 25), baseline characteristics of participants are shown in Table 1, and a flowchart of the study is outlined in Figure 1.

**TABLE 1:** Baseline characteristics of the exergaming and control group.

	Exergaming (*N* = 27)	Control (*N* = 25)
Age (years)	37 (8)	34 (9)
Female (*N*)	13(48%)	12(48%)
BMI (kg/m^2^)	26.7 (4.1)	27.1 (5.0)
Body fat (%)	29.8 (7.7)	28.8 (6.4)
V̇O_2peak_ (mL⋅kg^−1^⋅min^−1^)	39.9 (7.6)	40.6 (7.2)
MVPA (min⋅day^−1^)	87.0 (63.6)	70.8 (40.0)
Media usage (h⋅day^−1^)	4.4 (1.9)	4.8 (2.4)
Computer gaming (h⋅day^−1^)	1.1 (1.6)	1.4 (1.6)

*Data are mean (SD) or N (%). Body mass index (BMI), peak oxygen uptake (V̇O_2peak_), moderate-to-very vigorous-intensity physical activity (MVPA).*

### Between-Group Differences

**FIGURE 2:**
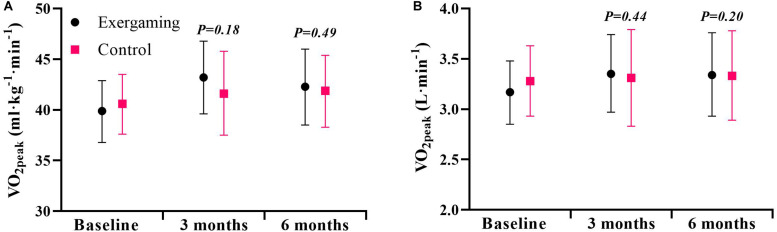
Cardiorespiratory fitness at baseline, 3 and 6 months for exergaming and control. **(A)** Peak oxygen uptake (V̇O_2peak_) expressed as mL⋅kg^−1^⋅min^−1^ and **(B)** V̇O_2peak_ expressed as L⋅min^– 1^. Data are mean, 95% CI. *P-*values are for the effect estimate (difference) from the linear mixed model.

**FIGURE 3:**
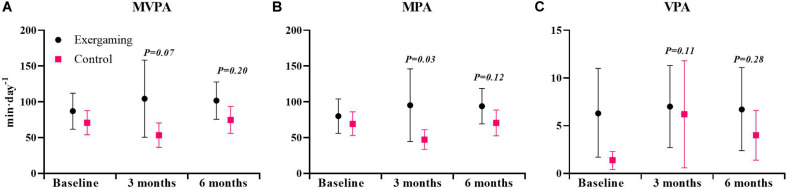
Physical activity level at baseline, 3 and 6 months for exergaming and control. **(A)** Moderate-to-very vigorous intensity physical activity (MVPA), **(B)** moderate-intensity physical activity (MPA), and **(C)** vigorous-intensity physical activity (VPA). Physical activity expressed as minutes per day (min⋅day^−1^). Data are mean, 95% CI. *P-*values are for the effect estimate (difference) from the linear mixed model.

For the primary outcome V̇O_2peak_ at 6 months, exergaming showed no significant benefit over control with a between-group difference of −0.7 mL⋅kg^−1^⋅min^−1^ (95% CI: −2.7 to 1.3, *P* = 0.49) and −0.10 L⋅min^−1^ (95% CI: −0.25 to 0.05, *P* = 0.20). The between group-difference at 3 months of 1.3 mL⋅kg^−1^⋅min^−1^ (95% CI: −0.6 to 3.3, *P* = 0.18) and 0.06 L⋅min^−1^ (95% CI: −0.09 to 0.20, *P* = 0.44) was neither clinically nor statistically significant (Figure 2 and Table 2). With a between-group difference of 21.9 min⋅day^−1^ (95% CI: 2.2–41.5, *P* = 0.03), the exergaming group had a significant increase in moderate-intensity physical activity at 3 months compared to control (Figure 3B and Table 3). Although not reaching statistical significance, there was a trend toward an increase in moderate-to-very vigorous-intensity physical activity for exergaming compared to control at 3 months (*P* = 0.07) (Figure 3A and Table 3). At 6-months, these between-group differences were no longer present (Figures 3A,B and Table 3). For vigorous- and very vigorous-intensity physical activity, there were no significant between-group differences at neither 3 nor 6 months (Figure 3C and Table 3). We removed two extreme outliers for physical activity levels from the analysis, as these were likely due to measurement errors. As can be seen from Tables 4–6, there were no significant between-group differences for any of the other secondary outcomes at 3 or 6 months. For hs-CRP, we detected one extreme outlier that was removed from analysis, likely due to an infection unrelated to the intervention.

**TABLE 2:** Cardiorespiratory values at baseline, 3 and 6 months for exergaming and control group.

		Exergaming		Control			
Variable	Month	*N*	Mean (*SD*)	*N*	Mean (*SD*)	Difference (95% CI)	*P*
V̇O_2peak_ (mL⋅kg^–1^⋅min^−1^)	0	27	39.9 (7.6)	25	40.6 (7.2)		
	3	21	43.2 (7.9)	18	41.6 (8.3)	1.3(−0.6to3.3)	0.18
	6	16	42.3 (7.0)	19	41.9 (7.4)	−0.7(−2.7to1.3)	0.49
V̇O_2peak_ (L⋅min^−1^)	0	27	3.17 (0.80)	25	3.28 (0.84)		
	3	21	3.35 (0.85)	18	3.31 (0.96)	0.06(−0.09to0.20)	0.44
	6	16	3.34 (0.78)	19	3.33 (0.92)	−0.10(−0.25to0.05)	0.20

*Mean (SD) are descriptive data, and the effect estimate (difference) is from the linear mixed model. Peak oxygen uptake (V̇O_2peak_).*

**TABLE 3:** Physical activity at baseline, 3 and 6 months for exergaming and control.

		Exergaming		Control			
Variable	Month	*N*	Mean (*SD*)	*N*	Mean (*SD*)	Difference (95% CI)	*P*
MPA (min⋅day^−1^)	0	27	80.1 (60.5)	24	69.3 (39.7)		
	3	12	95.3 (79.9)	12	47.3 (21.6)	21.9(2.2−41.5)	0.03
	6	18	93.9 (36.0)	18	70.6 (36.0)	13.3(−3.4to30.0)	0.12
VPA (min⋅day^−1^)	0	27	6.3 (11.8)	24	1.4 (2.3)		
	3	12	7.0 (7.2)	12	6.2 (8.8)	−3.9(−8.8to0.9)	0.11
	6	18	6.7 (8.8)	18	4.0 (5.1)	−2.3(−6.5to1.9)	0.28
VVPA (min⋅day^−1^)	0	27	0.6 (1.5)	24	0.1 (0.4)		
	3	12	1.6 (4.1)	12	0.3 (0.6)	0.7(−0.5to1.8)	0.26
	6	18	1.1 (2.4)	18	0.2 (0.7)	0.3(−0.7to1.3)	0.61
MVPA (min⋅day^−1^)	0	27	87.0 (63.6)	24	70.8 (40.0)		
	3	12	104.4 (84.8)	12	53.4 (26.8)	18.9(−1.2to38.9)	0.07
	6	18	101.7 (52.2)	18	74.8 (37.7)	11.1(−5.9to28.2)	0.20

*Mean (SD) are descriptive data, and the effect estimate (difference) is from the linear mixed model. Moderate-intensity physical activity (MPA), Vigorous-intensity physical activity (VPA), very vigorous-intensity physical activity (VVPA), moderate-to-very vigorous-intensity physical activity (MVPA).*

**TABLE 4:** Anthropometric values at baseline, 3 and 6 months for exergaming and control group.

		Exergaming		Control			
Variable	Month	*N*	Mean (*SD*)	*N*	Mean (*SD*)	Difference (95% CI)	*P*
Body mass (kg)	0	27	80.6 (15.1)	25	82.1 (19.4)		
	3	21	77.9 (13.7)	18	79.3 (17.3)	−0.6(−2.5to1.4)	0.57
	6	19	78.8 (13.8)	20	80.6 (17.7)	−0.9(−2.8to1.1)	0.38
BMI (kg/m^2^)	0	27	26.7 (4.1)	25	25.8 (4.3)		
	3	21	25.5 (2.9)	18	25.2 (4.1)	−0.2(−0.9to0.4)	0.45
	6	19	25.5 (2.9)	20	25.4 (4.0)	−0.4(−1.0to0.3)	0.25
Body fat (kg)	0	27	24.2 (8.7)	25	23.9 (8.0)		
	3	21	21.4 (6.0)	18	22.4 (7.0)	−0.3(−1.8to1.3)	0.75
	6	19	20.9 (5.6)	20	22.0 (6.6)	−0.5(−1.0to0.3)	0.55
Body fat (%)	0	27	29.8 (7.7)	25	28.8 (6.4)		
	3	21	27.6 (6.7)	18	28.0 (6.1)	−0.2(−1.7to1.2)	0.76
	6	19	26.7 (6.3)	20	27.2 (5.6)	−0.4(−1.8to1.0)	0.59
Visceral fat (cm^3^)	0	27	105.5 (33.2)	25	102.5 (33.9)		
	3	21	94.0 (25.7)	18	97.2 (31.4)	−1.6(−8.5to5.3)	0.64
	6	19	92.1 (23.3)	20	96.7 (29.0)	−3.6(−10.5to3.2)	0.29
WHR	0	27	0.95 (0.07)	25	0.96 (0.09)		
	3	21	0.92 (0.06)	18	0.94 (0.07)	−0.01(−0.03to0.01)	0.44
	6	19	0.93 (0.06)	19	0.94 (0.06)	0.00(−0.02to0.02)	0.91

*Mean (SD) are descriptive data, and the effect estimate (difference) is from the linear mixed model. Body mass index (BMI), waist-to-hip ratio (WHR).*

**TABLE 5:** Resting variables at baseline, 3 and 6 months for exergaming and control.

		Exergaming		Control			
Variable	Months	*N*	Mean (*SD*)	*N*	Mean (*SD*)	Difference (95% CI)	*P*
SBP (mmHg)	0	27	118 (11)	25	116 (11)		
	3	18	121 (13)	17	117 (12)	3(−2to7)	0.26
	6	19	115 (10)	19	112 (9)	1(−3to6)	0.55
DBP (mmHg)	0	27	79 (8)	25	76 (9)		
	3	18	81 (10)	17	77 (11)	1(−4to5)	0.80
	6	19	76 (5)	19	74 (9)	−1(−5to3)	0.68
RHR (beats⋅min^−1^)	0	26	61 (10)	25	61 (10)		
	3	18	64 (10)	17	63 (9)	2(−3to7)	0.46
	6	19	61 (10)	19	59 (9)	2(−3to6)	0.49

*Mean (SD) are descriptive data, and the effect estimate (difference) is from the linear mixed model. Systolic blood pressure (SBP), diastolic blood pressure (DBP), resting heart rate (RHR).*

**TABLE 6:** Blood variables at baseline and 6 months for exergaming and control.

		Exergaming		Control			
Variable	Months	*N*	Mean (*SD*)	*N*	Mean (*SD*)	Difference (95% CI)	*P*
Gluc-0 (mmol⋅L^−1^)	0	24	4.9 (0.3)	24	4.8 (0.5)		
	6	17	5.0 (0.5)	19	4.8 (0.4)	0.1(−0.1to0.3)	0.24
Gluc-120 (mmol⋅L^−1^)	0	24	5.8 (1.9)	24	5.3 (1.2)		
	6	17	5.3 (0.9)	18	5.6 (1.0)	−0.1(−0.7to0.6)	0.85
Gluc-tAUC (mmol⋅L^−1^)	0	24	808.4 (157.3)	24	755.0 (130.6)		
	6	17	761.6 (141.8)	18	729.2 (116.8)	−21.0(−143.6to101.5)	0.73
HDL-C (mmol⋅L^−1^)	0	24	1.31 (0.34)	24	1.31 (0.34)		
	6	17	1.27 (0.28)	19	1.24 (0.28)	0.04(−0.06to0.14)	0.39
LDL-C (mmol⋅L^−1^)	0	24	2.9 (1.0)	24	2.8 (0.8)		
	6	17	2.9 (0.7)	19	2.9 (0.7)	−0.3(−0.7to0.1)	0.12
Tot-C (mmol⋅L^−1^)	0	24	4.4 (0.9)	24	4.4 (0.8)		
	6	17	4.2 (0.7)	19	4.3 (0.6)	−0.2(−0.6to0.2)	0.33
Triglycerides (mmol⋅L^−1^)	0	24	1.01 (0.52)	24	1.05 (0.46)		
	6	17	0.88 (0.36)	19	0.92 (0.34)	0.00(−0.26to0.25)	0.98
HsCRP (mg⋅L^−1^)	0	24	1.51 (1.12)	24	1.87 (1.95)		
	6	16	0.89 (0.61)	19	1.23 (1.04)	0.13(−0.99to1.15)	0.79

*Mean (SD) are descriptive data, and the effect estimate (difference) is from the linear mixed model. Fasting glucose (Gluc-0), glucose 120 min post glucose loading in oral glucose tolerance test (Gluc-120), total area under the plasma glucose curve (Gluc-tAUC), HDL-Cholesterol (HDL-C), LDL-cholesterol (LDL-C), high-sensitivity c-reactive protein (HsCRP).*

### Exergaming Group

Participants in the exergaming group performed on average 12 (Range: 0–42) exergaming sessions during the intervention period (Table 7). Table 7 shows exercise intensities and perceived enjoyment and exertion for the exergaming group.

**TABLE 7:** Exergaming frequency, duration, intensity with perceptual and affective responses for exergaming participants (*N* = 27).

	Mean (*SD*)
Total sessions (*N*)	12 (13)
Sessions 3 months (*N*)	10 (9)
Sessions/week – 6 months *(N)*	0.5 (0.4)
Sessions/week – 3 months *(N)*	0.7 (0.6)
Exergaming – 6 months (min⋅week^−1^)	25.8 (23.2)
Exergaming – 3 months (min⋅week^−1^)	36.3 (35.4)
Exergaming – 6 months (min)	632.6 (740.0)
Exergaming – 3 months (min)	506.0 (538.1)
Exergaming duration (min⋅session^−1^)	46.1 (8.9)
Feelings Scale	3 (1)
PACES	99 (10)
Borg	5 (1)
Average HR (%)	75.4 (7.5)
Max HR (%)	89.4 (4.9)

*Data are mean (SD). Physical Activity Enjoyment Scale (PACES), Heart Rate (HR) expressed as a percentage of heart rate peak.*

Due to the large variation in exergaming frequency, we used multiple regression to predict gaming frequency from gender, age, moderate-to-very vigorous physical activity, V̇O_2peak_, and digital game usage at baseline. None of the five variables alone (adj. *R*^2^ = -0.039 to 0.077, *P* = 0.09–0.90) or together (adj. *R*^2^ = -0.040, *P* = 0.56) could significantly predict gaming frequency. In addition, further analysis was undertaken to assess how exergaming frequency and exergaming intensity affected changes in primary and secondary outcomes at 6 months. Neither primary or secondary outcomes could be predicted by the multiple regression model (adj. *R*^2^ = -0.320 to 0.285, *P* = 0.12–0.96).

## Discussion

The main findings of this study were: (1) providing inactive adults with free access to a biking exergaming platform for 6 months lead to no improvements in V̇O_2peak_ or other health benefits, compared to a control group without such access; (2) at 3 but not 6 months the exergaming group had increased their physical activity levels more than the control group; and (3) although exergaming participants rated the enjoyment of exergaming high, exergaming frequency was low.

### Cardiorespiratory Fitness

To our knowledge, this is the first sufficiently powered long-term study assessing the effects of providing inactive adults with access to an exergaming platform on cardiorespiratory fitness. Our findings showing no between-group differences in V̇O_2peak_ between the exergaming group and the control group at either 3 or 6 months are thus novel. Our results are in line with a previous study where 3-months access to an exergame did not result in any significant change in V̇O_2peak_ (Owens et al., 2011). The present study extends these findings by being appropriately powered and with a longer follow-up period. Furthermore, our results are in line with a 12-month unsupervised HIIT intervention, where participants were able to choose from several HIIT protocols, which also failed to demonstrate beneficial effects on V̇O_2max_ (Roy et al., 2018). Effectiveness of an exercise program to induce changes in V̇O_2peak_ is dependent upon the intensity, duration, and frequency of exercise (Wenger and Bell, 1986). Intensity reached during exergaming sessions in the present study is well above the minimum threshold for developing cardiorespiratory fitness, even when considering that heart rate measurements overestimate exercise intensity in exergaming (Swain and Franklin, 2002; Berg and Moholdt, 2020). Also, the duration per exergaming session fulfils The American College of Sports Medicine’s (ACSM’s) recommendations to enhance V̇O_2peak_ (Garber et al., 2011). Therefore, the lack of improvement in V̇O_2peak_ in the exergaming group in our study is likely explained by the observed low exergaming frequency. Although ACSM recommends aerobic exercise to be performed 3–5 days per week, it has previously been shown that when HIIT is performed to exhaustion, just one weekly exercise session may suffice to improve V̇O_2peak_ (Nakahara et al., 2015). However, in the present study, only three participants averaged more than one exergaming session per week throughout the intervention. Previously, the premise has been that a high enjoyment of exercise will lead to increased participation and adherence (Salmon et al., 2003; Williams et al., 2008). However, the results from this study show that although participants rated the enjoyment of exergaming high, this was not enough to stimulate exergaming frequency levels that could induce changes in V̇O_2peak_. Future studies should assess the short- and long-term effects of using this exergaming platform regularly and determine factors that can motivate users to start and continue using the exergaming platform.

### Physical Activity Level

In the current study, we observed a significant between-group difference in moderate-intensity physical activity at 3 months, during the intervention, but not at 6 months, post-intervention. This finding indicates that when previously inactive adults have access to the present exergaming platform, their physical activity level increases compared to a control group, but that activity levels are not sustained when access to the exergame is removed, suggesting no transfer effect. Our findings that physical activity increased in the exergaming group midst intervention, at 3 months, are in line with findings that have shown exergaming interventions using bicycles to create greater adherence compared to stationary cycling alone (Warburton et al., 2007; Rhodes et al., 2009). Our study extends these findings by not only assessing exergaming frequency but by objectively assessing physical activity. Furthermore, in our study, participants self-selected how often and for how long they wanted to exercise, increasing validity in a free-living situation. Our findings showing a short- but not long-term increase in physical activity is in line with recent findings on Pokémon Go (Althoff et al., 2016; Howe et al., 2016). However, the lack of between-group differences in any measure of physical activity at 6 months in our study stands in contrast to a recent study which concluded that exergaming increased physical activity both during the last week of the intervention, 1-week post-intervention and at 6-month follow-up (Bock et al., 2019). However, findings in the study by Bock et al. (2019) are limited by relying on self-reported physical activity since those enrolled in an intervention group may over-report physical activity due to expectations (Staiano et al., 2017). Another possible explanation for the contrasting findings between the present study and the aforementioned study by Bock et al. (2019) was the lack of access to the exergaming platform after the intervention period in our study, whereas participants in the exergaming group in the study by Bock et al. (2019) had access to the exergame also during the follow-up period. This highlights the need to assess how accessibility affects long-term adherence to exergaming. We speculate that the reasons for why exergaming participants were unable to sustain the increased physical activity levels compared to control also at the 6-month assessment were; (1) participants viewed exergaming as entertainment rather than exercise, therefore these effects wore off once the intervention stopped or (2) the low exergaming frequency; participants did not observe any positive health benefits from exercise and consequently had less motivation to continue. However, this needs to be addressed in future studies, both from a qualitative and quantitative perspective.

### Body Composition and Markers of Cardiometabolic Health

We found no benefits of exergaming on body composition, resting blood pressure, or blood variables at either 3 or 6 months. Results from previous exergaming interventions are inconsistent (Street et al., 2017; Bock et al., 2019). It is reasonable to suggest that exergaming frequency was too low to confer improvements in any marker of cardiometabolic health, like what we discussed as the reason for no improvement in V̇O_2peak_. Indeed, a previous study showed that HIIT performed three, but not two, times per week improved total cholesterol, LDL-C, and body-fat percentage in inactive but otherwise healthy adults (Stavrinou et al., 2018). Of note, both blood pressure and circulating blood markers were within the normal range at baseline in our study, which could limit potential beneficial effects from exercise. Although participants in the present study could be classified as overweight (average BMI > 25 kg/m^2^), exercise volume in the exergaming group was likely too low to induce changes in body composition (Friedenreich et al., 2015; Sultana et al., 2019).

### Limitations

There are some limitations to our study. Primarily, due to the low exergaming frequency we were unable to assess the effectiveness of regular exergaming for improving cardiorespiratory fitness and health. Our findings are also limited by a relatively high drop-out rate. In our sample-size calculation, we had anticipated a 15% drop-out rate, but for the 6-month testing only 35 of the included 52 participants showed up for testing of the primary outcome, V̇O_2peak_, corresponding to a 30% drop-out rate. Of note, two of the exergaming participants with the highest exergaming frequency (28 and 35 exergaming sessions respectively) dropped out from the V̇O_2peak_ assessment at 6 months. Also, in the present study, we exclusively assessed physiological outcomes and cannot exclude that participants in the exergaming group could have improved cognitive abilities or had psychological benefits. Furthermore, exergaming frequency may have been limited by the logistics of only having 4 available exergaming platforms and the fact that they were kept behind a locked door at the St. Olav’s University Hospital hindering the participants to drop-in for exergaming sessions without pre-arranging. Finally, although the SenseWear armband has been validated, it tends to underestimate vigorous exercise in general and particularly during cycling exercise (Koehler and Drenowatz, 2017). This might have influenced the physical activity measures, especially at the 3-month assessment.

## Conclusion

Our study indicates that merely offering inactive adults access to an exergaming platform for 6 months was insufficient to change exercise behavior and thus improve cardiorespiratory fitness or any of the other health outcomes we measured. Even if the participants in our study rated the exergaming as enjoyable, the frequency of exergaming was low. Future work should focus on assessing the effects of regular exergaming on cardiorespiratory fitness and determine how best to implement such exercise training outside the laboratory, e.g., in a home setting to increase retention and adherence.

## Data Availability Statement

The raw data supporting the conclusions of this article will be made available by the authors, without undue reservation.

## Ethics Statement

The studies involving human participants were reviewed and approved by The Regional Committee for Medical and Health Research Ethics in Central Norway. The patients/participants provided their written informed consent to participate in this study.

## Author Contributions

JB, TM, and AW were involved in the study design. JB collected the data and wrote the manuscript draft. JB, TM, and SL have analyzed the data. TM, AW, and SL revised the manuscript. All authors contributed to the article and approved the submitted version.

## Conflict of Interest

The Playpulse exergaming platform was an NTNU innovation. The inventors have started a spin-off company. AW has no official role in the company, but he owns some shares in Playpulse, which could be construed as a potential conflict of interest. The remaining authors declare that the research was conducted in the absence of any commercial or financial relationships that could be construed as a potential conflict of interest.
